# An Attempt to Determine with Precision Such Injuries of the Head as Necessarily Require the Operation of the Trephine

**Published:** 1794

**Authors:** Sylvester O'Halloran

**Affiliations:** M.R.I.A. Honorary Member of the Royal College of Surgeons in Ireland, and Surgeon to the County of Limerick Hospital.


XVI. *An Attempt to determine with Precifion fuch Injuries of the Head as neceffarily require the Operation of the Trephine. By* Sylvefter O'Halloran, *Efq. M.R.I.A. Honorary Member of the Royal College of Surgeons in Ireland, and Surgeon to the County of Limerick Hofpital. From the Tranfactions of the Royal Irifh Academy,* Vol. IV.

Non fingendum, aut excogitandum, fed inveniendum, quid natura faciat aut ferat. BACON.

WERE we to eftimate the lights thrown on particular diforders by the number of eminent men who have treated of them, we fhould conclude that thofe produced by external injuries of the head muft be beft underftood, as from the days of Hippocrates to our own they have been confidered with moft particular attention. But however great our obligations to our anceftors, and to many illuftrious and learned moderns and contemporaries may be, yet our knowledge of this fubject, fo interefting to mankind, is ftill incomplete. The various

 diforders diforders fubfequent to injuries on the head have by no means been difcriminated with fufficient precifion; nor have their fymptoms or modes of treatment been clearly afcertained; even the ufe of the trepan is now more indefinite than it was a century ago. The late Mr. Pott, as diligent and accurate an obferver as any age or nation has produced, complains "of the darknefs and obfcurity of this part of "furgery."

Enabled by clofe and diligent obfervations, and by extenfive practice in this line for above thirty years, I have ventured, under the aufpices of the Academy, to determine a queftion, perhaps the niceft and moft involved in furgery.

The operation of the trepan is very ancient; but being confined to narrow limits, was feldom performed without the greateft caution and circumfpection; for it was interdicted over or near the futures, near the os fquamofum, and on or very near the occiput. This well explains an anecdote in our early hiftory; for Connor Mac Neaffa, King of Ulfter, that generous protector of the literati of his days, and cotemporary with Julius Cæfar, having his fkull fractured in battle, his firft furgeon, Finighin, refufed to apply the trepan till his fafety was guaranteed by the nobles of the country, in cafe it did not fucceed.

But the experience of the laft and prefent centuries has proved, that there are very few parts of the head on which the trephine may not be applied when neceffary. However, this information, in itfelf fo interefting, has by no means anfwered the ends that fhould be expected from it; for inftead of our determining, or even attempting to limit the cafes to which it fhould be confined, it has been fince adopted in almoft every fevere injury of the head, accompanied with untoward fymptoms. To point out with perfpicuity and precifion the cafes in which it can alone be ufeful to the patient, is the object of this paper. Thefe obfervations may be arranged under two articles—Fractures of the Cranium, and depofits on the furface of the Brain or on its Membranes. Yet, even in the cafe of fractures, long experience has convinced me that many of them require no operation. As this is an object of great moment, I fhall be as clear and concife as poffible. To this purpofe, I have felected three out of a great number of cafes; and the rather becaufe each had its particular fymptoms, though all tending to the fame point.

Cafe I. Mrs. Grogan fell from a window into the ftreet, and received a violent contufion on the front of the coronal bone. I faw her the next morning, and found a confiderable tumour, which to the touch feemed to contain fome fluid; but as I had feen many fimilar ones fubfide in four or five days, by the ufe of compreffes wet in fpirits, I treated this in the fame manner. The fwelling, however, remained, and in five days I propofed opening it, which fhe would not permit. The fixth day fhe again fent for me; the tumour was ftill the fame, but the fluctuation not fo fenfible. I laid open the part, and a good deal of coagulated blood was difcharged. She complained all that and the next day of pain, and a thin bloody fanies came from the wound. I found not only the bone bare, but a confiderable fracture, with fome depreffion. I preffed with my finger the fides of the bone, but it remained firm to the touch, and fhe felt no uneafinefs from it. I kept the wound open for fome days, and finding no alarming fymptoms, fuffered it to heal, which it did by the end of the month. I recommended her to keep the part covered for fome time, on account of the thinnefs of the cicatrix. This fhe neglected. In fome days after, leaning over a gara garden wall, with a ſmart wind in her face; ſhe was ſeized with a violent pain, and imagined the wind was piercing into her head. She ſlept little, and was the whole night in a fever. Next morning I found the forehead greatly ſwelled, and let out a conſiderable quantity of matter. In ſome time it got well; ſhe had it covered with adheſive plaiſter, and never after complained.

Caſe II. Samuel Haſte received a wound on the upper part of the right parietal bone, of two inches long, with a looſe bone and fracture. Though the fracture could not admit of a doubt, yet there ſeemed to be but little depreſſion, and the ſides of it were firm to the touch. I ſaw no reaſon for the operation at preſent, but carefully attended to the ſymptoms. In the ſpace of four weeks the bone became covered, the wound healed, and he has ſince enjoyed perfectly good health.

Caſe III. M'Namara received a wound on the forehead, and near the left ſide of the frontal ſinus. In a few days after he was brought to the hoſpital; his pulſe was full, and he complained of a great head-ach. Upon examining through a ſmall aperture, I perceived the bone bare and rough, and concluded there was a fracture. I removed the integuments, and found the bone fractured near two inches in length, but still the parts were nearly upon an equality, and the bone firm to the touch. As he bled a good deal I did not direct venæsection, but ordered some powders of nitre and jalap, and the saline mixture. His head-ach was not abated next morning, so I directed a large blister to his back, which I desired should remain on for twenty-four hours. This removed the head-ach, and the wound went on well. In five days after, he complained of violent shiverings, and I should have concluded them to be the precursors of matter forming on the brain, had I, in the course of many hundred fractured skulls, seen a similar instance; but I had not. He was not trepanned; the bark, in some time, removed these complaints, and he is again abroad.

Having thus, I apprehend, clearly proved, that many fractured skulls do not demand the trepan, it remains that I should clearly discriminate between those and other seemingly slight fractures, which absolutely require it. In the course of my practice I have constantly observed, that fractures of the cranium are more extended in the inner than the outward table, table, and of courſe, that ſimple fractures, ſuch as I have deſcribed, may do conſiderable damage within, whilſt all is fair without. Fractured ſkulls in general (with a very few exceptions) are attended with no very alarming ſymptoms for many days. They are free from fever and inflammation; nor is reaſon in the ſmalleſt degree impaired. But in the courſe of ten, twelve, or fifteen days, if any preſſure reſt on the brain, the patient becomes heavy, grows drowſy, comatoſe, and ſometimes convulſed. The firſt appearance of any of theſe ſymptoms is the critical time of alarm, and the operation ſhould be immediately proceeded to.

Caſe IV. J. O'Mara received a violent blow over the middle of the left parietal bone. It was for a good many days dreſſed by a perſon in the neighbourhood, but not appearing to mend, I was applied to. I found a ſeemingly ſlight contuſed wound, with a bare bone; and ſeeing him heavy and drowſy, I concluded a preſſure on the membranes of the brain. As he appeared more ſtupid the next morning, I removed the ſcalp, and found underneath a conſiderable depreſſion. The operation immediately followed the inciſion; the depreſſed part was raifed, fome fmall fplints removed, and he is, at this day, a healthy man.

Cafe V. William O'Neil received a very extended wound on the fuperior part of the left parietal, which bared the bone for near two inches, with a violent contufion about the center of that bone. The wound was dreffed, and every precaution taken to prevent fever and inflammation. He went on tolerably well for twelve days, except that the wound on the parietal never exhibited a pleafing afpect. About this time I perceived him to grow heavy and drowfy. I more narrowly infpected the head: but though the bare bone became fenfibly difcoloured, and though I expected exfoliation would follow, yet I was well convinced that no fracture was there. I interrogated him clofely. He told me the leaft noife difturbed him, and he imagined that found was conveyed through the fide of his head, as well as through his ears. I now more narrowly infpected the contufion on the fide of the head, which had hitherto been dreffed with a comprefs dipped in fpirits only. I thought I felt an obfcure fluctuation: at any rate the fymptoms determined me to open this part. But what was my furprife when I found the bone underneath not only only fractured, but beat into ſmall pieces, at the point of percuſſion. I trepanned on the ſpot; removed bits of bone, and raiſed others to their level. After this every thing went on well, except the wound at top, which threw off ſeveral exfoliations, and remained open many days after the fractured parts healed. This man became as ſtout and well as poſſible.

After theſe proofs, that even in many fractured ſkulls the operation of trepanning may be diſpenſed with, what pretence can we offer for trepanning in wounds of the cranium inflicted with inciſive inſtruments? I know of none that can juſtify ſo violent an outrage to nature, except ſymptoms of extravaſation appear, which I believe very ſeldom happen where the ſkull itſelf is injured.

Caſe VI. Edward Power received a deſperate wound of a back ſword, extending from the top of the coronal bone to the orbit of the left ſide, forming an extended and frightful chaſm, in which were included the bone, membranes, and brain. It bled conſiderably, as may well be ſuppoſed. He remained expoſed to the open air for near three hours after, and had not ſo much as a bit of rag to cover it. Fever and inflammation of the brain might reaſonably

ſonably be apprehended; yet, by a couple of bleedings, and ſome other antiphlogiſtics, the man was completely cured in five weeks, without exfoliation or the ſmalleſt operation. To this I ſhall add an inſtance given by La Motte*, of a man who received a blow of a ſabre on the right parietal bone, which divided the longitudinal ſinus and the left parietal, extending almoſt from ear to ear; and yet this man recovered without any operation.

Having diſpoſed of the above very intereſting queſtions, we ſhall now remark on ſuch fractures as neceſſarily require the operation in the firſt inſtance. Theſe are fractures accompanied with depreſſion, with or without wounds. For, not to advert to the reſtraint ſuch preſſure muſt neceſſarily cauſe to the motion of the brain, ſufficient in itſelf to produce fatal effects, if the depreſſed parts be pointed and ſharp, as is moſtly the caſe, acting againſt the uniform pulſation of the brain, the membranes muſt in ſome time be cut through, and the brain itſelf wounded; and whilſt this tragic work is going on, we have no ſymptoms to indicate the approaching danger till it is paſt remedy. The

* Traité complet de Chirurgie, t. iii, p. 343.

two two following inftances contrafted will explain and juftify this pofition.

Cafe VII. A girl about feven years of age received a fevere fracture, with profound depreffion, on the left parietal bone; the integuments were entire, the girl quite compofed and fenfible, but the depreffion was fo deep that it could contain a very fmall egg. Such was her fituation when brought to me, half an hour after the injury. Seeing that it would require three or four crowns of the trephine to raife this extended fracture, I requefted of Mr. Wallace, a military furgeon, and Mr. Pierce, to affift me in this charitable work. I removed all the integuments, wiped away the blood, and whilft thefe gentlemen with their fingers made compreffions over the bleeding veffels, I began to operate on the inferior parts of the bone. I then commenced a fecond on the upper part, and in a line with this; but the two elevatois, though acting at the fame time, had no effect on the depreffion. Two more crowns were then applied to the fides of the bone, and parallel to each other. Four levers acting in conjunction, it aftonifhed me to fee with what a fudden fpring the depreffed parts refumed their former ftation. Notwithftanding the great extenfion extenſion of this fracture, the loſs of covering, and of the bone itſelf, by four crowns of the trephine, this girl never after had the ſmalleſt untoward ſymptom.

What muſt have been the event of this caſe if not ſpeedily relieved, the following will ſhow.

Caſe VIII. Patrick Caſey, aged about eighteen, was thrown from his horſe with great force; the conſequence was a fracture in part of the coronal bone, with a conſiderable depreſſion. I was requeſted next morning to viſit him; and ſeeing his ſituation, I was juſt proceeding to the operation, when a ſurgeon of the city appeared, who ſaid he was employed by Caſey's maſter to attend to him. The depreſſion was ſo conſiderable, that the lower edge of the fracture was beaten under the uninjured part; here I intended my operation, in order the more ſpeedily and effectually to diſengage the fracture. But this was oppoſed; it was obſerved, that trepanning ſo low down would leave a great deformity, and that the end propoſed would be as well anſwered by perforating the bone at top. I oppoſed it in vain. I ſaw that the friends of the boy, who were preſent, as well as himſelf, wiſhed to have it done ſo. I trepanned, introduced the elevator, but could make make no impreſſion, as the depreſſed parts were beyond the reach of the inſtrument. I now propoſed a ſecond opening on the lower edge of the fracture, the firſt being impoſſible to anſwer the end propoſed. This was not agreed to. It was obſerved, that an opening being made, no depoſit could be formed, and that the depreſſed part would become gradually detached, and probably come away, which has ſometimes happened. The ſore was carefully dreſſed, but the dura mater never aſſumed a right aſpect; however his ſpirits were good, and he had no complaint but what aroſe from the ſore itſelf. The diſcoloration of the dura mater made me try, on the 15th day, and again on the 17th, the effects of the elevator, but in vain. He was up every day. The 24th, looking out of a window for ſome time, he perceived a ſlight chillineſs; at bed-time he grew hot and feveriſh, was very reſtleſs, and had a ſtrong ſhaking fit. This alteration was aſcribed to his making too free with himſelf. But I ſaw and dreaded the conſequences. I told the people that theſe alarming ſymptoms proceeded not from cold, but from the conſtant and uniform preſſure on the brain; and that if any chance remained for his recovery, which I much doubted, doubted, it muſt be by a ſpeedy removal of the cauſe; and if they conſented I would not ſhrink from this diſagreeable buſineſs. I directly made the ſecond perforation, and ſoon raiſed the part; but, alas! the miſchief had been already completed. That day and the next he ſeemed a good deal lighter: but, about ten at night of the ſecond day, his neck was obſerved to be covered by a bloody ichor iſſuing from the ſore. Next morning his pulſe was more languid, and the dura mater quite black. Towards night the bloody ichor increaſed; he became ſlightly convulſed, with ſtupor. About ten the ſubſtance of the brain poured forth, and he expired next morning.

Fractures with a depreſſed bone always require the operation; and though ſome caſes may be adduced where an happy recovery has ſucceeded without this adoption, yet it muſt ariſe from particular circumſtances by no means to be depended on. For inſtance, the depreſſion may be uniform, ſo that no point of the fracture may preſs on the brain. In ſuch an inſtance, no doubt things may come about without trepanning; but have we any ſymptom to determine this point? None that I know of. Numbers I have ſeen periſh by neglecting the operation, ration, becauſe they found themſelves free from pain and fever at the beginning. As, then, trepanning is a ſafe operation in any tolerable hands, no conſideration ſhould make the ſurgeon decline or procraſtinate it. He has already ſeen the happy conſequences of it when performed in the firſt inſtance. He will now ſee what may be hoped for from nature, even in the moſt deplorable caſes, from neglect or delay.

Caſe IX. I was ſent for to Patrick Kelly, who had received repeated blows on the left parietal bone, which produced a very extended fracture, with a ſlight contuſed wound of the integuments. He had been attended for ſome time by a young man in the neighbourhood. In the courſe of about ten days he became heavy and drowſy; the complaints increaſed, and when I was ſent for he was comatoſe, languid, and oppreſſed; ſo much ſo, that I apprehended any operation uſeleſs, and had ſome thoughts of immediately returning. But reflecting on the great reſources of nature, and that it would be in ſome ſort criminal not to diſcharge my duty, I removed the integuments on the interior part, where the depreſſion was greateſt, and directly applied the largeſt crown

I had.

I had. On removing the piece I introduced the elevator, raifed the depreffed parts, and was fatisfied, from the extent of the injury, that many pieces of bone would come away. Immediately after this he opened his eyes, knew me, and fpoke. I left directions for managing the dreffings, and ordered fome medicines of the nervous tribe. In eight days after I again vifited him, and found a confiderable part of the bone loofe. I made a flight incifion over it, and extracted it with my forceps. The next day he found a weaknefs of the neck and arm of the oppofite fide, and by night it attacked that entire extremity. In a few days after two more pieces of bones were removed, and again another. He laboured under this partial paralyfis for about fifteen days, and then gradually recovered.

Cafe X. I vifited Patrick Hayes, who, thirteen days before, had received a blow which had made a profound depreffion on the pofterior and near the fuperior part of the right parietal bone, and very near its junction with the occiput. It was attended with no wound, but a fmall perforation fcarce fufficient to admit the point of a probe. In fome days fymptoms of a depreffed bone came on. I found the man with with a ſlow, weak, but regular pulſe. He was quite comatoſe, and could not articulate. Upon paſſing a fine probe through the little perforation, I found the bone had been craſhed into ſmall bits, and was for ſome time at a loſs what to do, on account of the ſituation of the man and the nature of the injury. I declared to his friends that I ſtrongly apprehended the caſe mortal, but would do what depended on me if they conſented. Upon removing the ſcalp, the bone had been ſo far beat in that I concluded I ſhould find the membranes cut through. Could I raiſe any part of the fracture I ſaw to a certainty I could remove the whole, the injury being confined to the circumſcribed part. With my forceps, probe, and elevator, ſucceeding each other, I was fortunate enough to remove one ſplint. This afforded me more room; and ſo, by degrees, and with ſome patience, I cleared the dura mater of all incumbrance, without recurring to the trephine. It was greatly depreſſed, and though wounded in two or three places by the point of the bone, yet no where cut through, but diſcoloured and blackiſh. He opened his eyes after this, and appeared lighter. Antiſeptics and the bark were not omitted, as well as a generous diet; that is to ſay, ſtrong ſeaſoned broths, ſago, or gruel with wine, and wine whey; at times. In ſome time the dura mater roſe to its natural height, the diſeaſed parts ſloughed off, it gradually aſſumed its natural colour, and he recovered.

The next ſpecies of injury that requires trepanning is, depoſits of matter on the membranes of the brain, or on its ſurface. On this very obſcure and truly lamentable malady, my eſteemed friend, Mr. Deaſe, of the Royal College of Surgeons, has thrown much light, in a late ingenious treatiſe *. It is beyond doubt a complaint of a moſt ſerious and alarming nature. In the courſe of many years practice, and painful obſervations, I cannot give myſelf credit for a ſingle cure I ever performed in this way, when the ſymptoms of depoſit were formed; and whether the patient was or was not trepanned, the ſcene cloſed with death! However, this ill ſucceſs I complain of ſhould not deter others; for in this caſe the operation is indiſpenſible; inſtances of recovery from it can be adduced; and when a practitioner makes a fair prognoſtic he cannot be cenſured.

* Obſervations on Wounds of the Head.

This complaint is moftly confined to wounds of the fcalp and pericranium, but particularly of the latter. The fymptoms of matter forming under the cranium commence generally about the eighth day, fometimes later, but feldom exceed the fifteenth : thefe are, ficknefs at ftomach, head ach, a fmart fever, and ftrong fhaking fit; the wound affumes a paler colour, the difcharge is thin and pale, and the pericranium becomes more and more detached from the fkull. Thefe are the unequivocal figns that matter is formed on the cerebrum, or on its covering; and in thefe cafes, however deplorable the event, there is no refource but in trepanning. It is a fact well known to perfons of extenfive practice, that though this matter begins under the immediate point of percuffion, yet that it extends much farther. To operate, then, to any effect, I would recommend the application of more than one crown of the trephine. For inftance, the firft perforation being made, I would immediately proceed to a fecond, including a fegment of the former circle in it, by which means the iffue of matter is more facilitated, and if it fhould be found neceffary to open the dura mater, it will be done with greater effect by extending the wound of it.

Concuſſions of the brain are generally ſuppoſed to require the trepan. Dionis, an able writer of the laſt century, judges that the loſs of ſenſe and memory, immediately ſucceeding a violent injury, are ſufficient motives to proceed directly to the operation, and he illuſtrates his poſition by a caſe in point*. Mr. Pott, though he very judiciouſly points out what can be effected by the operation, namely, the raiſing of depreſſed bones, or the iſſue of blood or matter, yet he becomes an advocate for it on ſtupors immediately following a hurt. "For "though it may be reſolved into ſymptoms of "concuſſion," ſays he, "yet extravaſation "may ſo ſpeedily follow the firſt ſhock, as to "carry all the appearance of the firſt, whilſt "the ſecond is the real cauſe." But to a certainty extravaſations of blood, matter, or water, (for I have met with them all) do not immediately, nor for ſome days after, produce ſtupor or inſenſibility. Fractures with conſiderable depreſſion, extravaſations, &c. ſhow no alarming ſigns for many days; but ſtupor *immediately* following

* Cours d'Operations de Chirurgie, p. 510.

ing a fall or hurt *is an unequivocal ſign of concuſſion, and of concuſſion only.* Not but that I have met with three caſes, and each of them mortal, where the ſymptoms of concuſſion immediately appeared, though in each the fracture and depreſſion were evident. But this only proves that in each the injury was ſo great that the yielding of the ſkull was not ſufficient to deſtroy the force of motion.

In caſes of death, after injuries of the head, where concuſſion was the cauſe, I have invariably obſerved the following appearances:—the pericranium and ſkull were injured; the dura mater adhered to the latter; there was rarely any extravaſation of blood, and this but ſlight, and out of the reach of any inſtrument. In a word, I could get no information, except that in thoſe who died ſoon after the accident I have ſometimes thought the brain did nor completely fill the cavity of the cranium. To this let me add, that inſtances can be adduced where leaps or falls from an high place, on hard ground, where the head has been far removed from the ſeat of the injury, have produced all the ſymptoms of concuſſion. I well know that many have been trepanned, and great cures boaſted of in theſe diſorders; but ſure I am, that whatever merit they might juftly claim, by endangering nature's endeavours and protracting recovery, they had none with refpect to the merit of the cafe.

I divide concuffions of the brain into three claffes. 1. Mortal ones. 2. Where there is recovery with infanity. And 3. Where there is perfect recovery. From what has been faid it is evident that I had early made up my mind with refpect to trepanning in this complaint: I have fingled out two ftriking cafes in point in fupport of my opinion.

Cafe XI. A gentleman was thrown from his horfe, and found fpeechlefs and fenfelefs; and in this condition was brought home. A phyfician was fent for, who immediately let blood; but finding the comatofe indifpofition continue a fracture was fufpected, and I was called upon. The integuments were very thin, he had been clofe fhaved, and I could not be well decided. After the moft critical inveftigation I was convinced there was no fracture; befides, his fymptoms were the reverfe of thofe attending a fracture. Bleeding, blifters, finapifms, &c. fucceffively fucceeded each other; but he gained no ground. He remained for ten days after in the fame fituation, with frequent moanings, and without being capable of uttering a word, though he took nourifhment, nourifhment, drink, and whatever was offered him. About this time dawns of reafon and fymptoms of convalefcence appeared, and in a very fhort time he was reftored to perfect fanity of mind and body, and lived for many years after.

Cafe XII. Mr. M. was thrown from his horfe, and pitched on the crown of his head on a ftone pavement. He alfo received a contufed wound from a kick of the horfe on a pofterior part of the right parietal bone, which denuded the bone. He was taken up fenfelefs and fpeechlefs. He was profufely bled, and fuffered other evacuations in the courfe of the two fucceeding days, without the fmalleft amendment I was then fent for; and from the above recital of the cafe was fatisfied that there was no fracture. The pulfe, as in fuch cafes, was flow and full; he moaned much, and frequently put his hand to his head. Experience had taught me that profufe evacuations anfwer no good purpofe in thefe maladies, fo I gave him medicines of the nervous tribe, with a cold infufion of the bark[a]; and for his diet, veal broth, beef tea, and fometimes wine whey. In two days after his pulfe became firmer, but his reftlefnefs and anxiety increafed towards evening. About one next morning he grew perfectly outrageous, ſo as with difficulty to be kept in his bed, and I was called up. On reflection, nothing ſeemed to me ſo proper to calm theſe ſymptoms as ſedatives. I ſent immediately to the apothecary's, gave him myſelf a doſe; and finding him calmer, in half an hour, I left him, with directions that at what time ſoever it returned the doſe ſhould be repeated. At five in the morning the medicine was again given. He remained compoſed, was much better, and viſibly clear in his intellects. In a word, I left him perfectly reſtored the third day after; the wound in the head healed in ſome days after, and he recovered.

From the preceding facts and obſervations the following propoſitions may be deduced.

1. That many fractures of the ſkull do not require the application of the trephine.

2. That ſome apparently ſlight fractures do abſolutely require its application; in ſuch caſes the inner table of the ſkull is generally more hurt than the outer, and bad ſymptoms do not ariſe till towards the end of a fortnight after the injury.

3. That fractures with depreſſion require the application of the trephine, and that from ſuch there have been ſome ſurpriſing recoveries,

4. That

4. That depofits of matter on the membranes or furface of the brain require the trephine, though it feldom proves fuccefsful.

5. That concuffion of the brain, characterifed by *immediate ftupor and infenfibility*, does not require the trephine, unlefs accompanied with evident depreffion of the fkull or extravafation, neither of which produce bad fymptoms for fome days after the accident which has given rife to them.

